# Generation and characterization of tissue-type plasminogen activator transgenic rats

**DOI:** 10.1007/s11239-017-1582-1

**Published:** 2017-11-22

**Authors:** Yusuke Ito, Kengo Noguchi, Yoshiyuki Morishima, Kyoji Yamaguchi

**Affiliations:** 1 Rare Disease & LCM Laboratories, Daiichi Sankyo Co., Ltd., 1-2-58 Hiromachi, Shinagawa-ku, Tokyo, 140-8710 Japan; 2 Pharmacovigilance Department, Daiichi Sankyo Co., Ltd., Tokyo, Japan; 3 Medical Science Department, Daiichi Sankyo Co., Ltd., Tokyo, Japan

**Keywords:** Fibrinolysis, Thrombosis, Tissue-type plasminogen activator, Transgenic rat, Species difference

## Abstract

To address a species difference in the responsiveness to human recombinant tissue-type plasminogen activator (rt-PA) between rats and humans, tPA transgenic (Tg) rats were generated and characterized. In the rats, transcriptional regulation of tPA was designed under the control of the endogenous tPA promoter. There were no significant differences in hematological parameters between the tPA Tg and non Tg rats. Plasma tPA concentration was significantly increased and serum free PAI-1 was significantly decreased in the tPA Tg rats. Significant overexpression of tPA mRNA in five major organs was also confirmed in the tPA Tg rats. In contrast, the extent of tPA mRNA induction by pathophysiological stimuli (focal cerebral ischemia) was comparable in the two strains. Earlier increase in the plasma D-Dimer level was observed in the tPA Tg rats in a model of thromboembolism compared with the non Tg rats. On the other hand, there was no statistically significant prolongation of bleeding time in a rat model of bleeding between the two strains. rt-PA showed dose-related blood flow restoration in a rat model of thromboembolic stroke in the tPA Tg rats from a dose (1 mg/kg, i.v.) similar to clinical doses for human stroke patients. In conclusion, tPA Tg rats, in which tPA is overexpressed and endogenous fibrinolytic activity is enhanced without hemostatic abnormality, were generated. tPA Tg rats would be beneficial for the pharmacological and the toxicological evaluation of rt-PA and other various fibrinolytic enhancers.

## Introduction

Tissue-type plasminogen activator (tPA) is an endogenous activator of the fibrinolytic system to dissolve blood clots [1]. Human recombinant tPA (rt-PA, recommended standard dose of 0.9 mg/kg, intravenous [i.v.]) is approved for the treatment of ischemic stroke within 4.5 h of symptom onset [2], demonstrating fibrinolysis enhancement is one of the promising approaches for the treatment of thrombotic disorders [3]. In fact, other classes of fibrinolytic enhancers such as tPA derivatives [4, 5], plasmin derivatives [6], Stachybotrys microspora triprenyl phenols (SMTPs) [7], and activated form of thrombin-activatable fibrinolysis inhibitor (TAFIa) inhibitors [8–11] have been reported, and several clinical trials are ongoing. However, there have been few successful clinical trials in human patients, at least regarding stroke [12, 13].

One of the possible causes for the difficulties is that no reliable animal stroke or other thrombotic model exists to evaluate fibrinolytic agents for clinical use because of the different fibrinolytic activities between animals and humans. For example, it is well-known that effective rt-PA doses in rat stroke models (ie, 10 mg/kg, i.v.) are much higher than those for human patients [14–16]. This suggests a species difference in the responsiveness to the agent between rats and human [17]. Since rt-PA has been reported to have various biological effects beyond fibrinolysis, such as low-density lipoprotein (LDL) receptor related protein-dependent intracellular signaling that might lead to vascular leakage and hemorrhagic transformation [18–20], there might be drawbacks or misinterpretation in efficacy and safety of rt-PA when the higher dose is administered to conventional rats. Thus, there is a need to develop clinically relevant animal models of stroke in which dose response to rt-PA is closer to human stroke patients to evaluate efficacy and safety of rt-PA appropriately in non-clinical studies.

To circumvent the species difference in the responsiveness to rt-PA between rats and humans, genetic manipulation of fibrinolytic factors in rats is one of the rational approaches. To date, many transgenic (Tg) animals have been generated in which fibrinolysis-related genes are manipulated, such as PAI-1 knockout mice, α_2_-PI knockout mice, and neuron-specific tPA overexpression mice [21–23]. However, these animals have not been applied to circumvent the difference in the response to human rt-PA. In addition, there is no report on rats in which the fibrinolysis-related genes have been manipulated. Since the body size of rats is larger than that of mice, several more elaborate thrombotic disease models (such as stroke) are more easily created in rats compared to mice. Therefore, Tg rats for overcoming these limitations in pharmacological and toxicological studies of rt-PA would be of value.

In the present study, we aimed at generating novel rat tPA Tg rats that possess targeted profiles as follows: (i) pathophysiological response of fibrinolysis is conserved (a transgene is controlled by its endogenous promoter), (ii) their hemostasis is normal, and (iii) effective doses of rt-PA in a thromboembolic stroke are closer to those of human patients. To meet these criteria, the tPA Tg rats, in which transcriptional regulation of tPA is designed under the control of the endogenous tPA promoter, were generated using a bacterial artificial chromosome (BAC) clone and the Red/ET system [24]. In addition, the tPA Tg rats were characterized in terms of hematology, gene expression, coagulation and fibrinolysis parameters, and pathophysiological response, and these characteristics were compared with those of non-transgenic (non Tg) rats. Furthermore, we determined the dose-response of rt-PA in a rat model of thromboembolic stroke in the tPA Tg rats and the non Tg rats.

## Materials and methods

### Animal care

All experimental procedures were performed in accordance with the in-house guidelines of the Institutional Animal Care and Use Committee of Daiichi Sankyo Co., Ltd. The frequency of animal health monitoring by animal care personnel was twice/day in weekday and once/day in weekend. Microbial monitoring were also conducted using sentinel animals once every 2 months. Proper cares were conducted or directed by attending veterinarians if abnormal animals (distresses in drinking water, feeding, or breathing or other abnormal behaviors such as self-injury, or abnormal posture) were observed. All surgery was performed under sodium thiopental (100 mg/kg, intraperitoneal [i.p.], Mitsubishi Tanabe Pharma Corporation) or isoflurane (3.5% for induction and 1.5% for maintenance, Pfizer) anesthesia, and all efforts were made to minimize suffering. Humane endpoint was applied in disease model experiments when the subjected animals showed the abnormalities listed above. Intravenous sodium thiopental (100 mg/kg) was administered to euthanize animals if the humane endpoints were applied. The protocol was approved by the Institutional Animal Care and Use Committee of Daiichi Sankyo (Permit Number: A1502377).

### Animals

Male tPARecBACTg (tPA Tg) rats and non Tg rats were generated and systematized using a BAC clone and the Red/ET system at the Institute of Immunology Co., Ltd. In brief, the BAC clone CH230-396J3, whose sequence is derived from Brown Norway rat, containing the rat tPA gene from − 85.5 to + 85.2 kb was obtained from the BACPAC Resources Center (Children’s Hospital Oakland Research Institute). Nucleotide sequence of the rat tPA between the BAC clone was identical to that of publicly deposited one (accession number NC_005115.4 ranging from 74,098,263 to 74,122,897). An EcoRI cleavage site was created in the intron region between exon 5 and 6 in the BAC clone so that the tPA transgene could be distinguished from the endogenous tPA gene. Fertilized eggs were collected from female Wistar rats (Charles River Laboratories Japan, Inc.) administered pregnant mare serum gonadotropin, and human chorionic gonadotropin to induce superovulation for pronuclear injection. The recombinant BAC clone was injected into the eggs, which were transplanted into pseudopregnant Wistar females to generate Tg rats. The tPA fragment derived from exon 4 was labeled with ^32^P and used as a probe for detecting the tPA gene. Genotypes of the Tg rats were analyzed by Southern blot analysis of tail DNA samples to confirm the 1.0 kb restriction fragment showing tPA gene insertion. The copy number was also determined using serially diluted BAC construct as standards. The male tPA Tg and the non Tg rats were maintained at the Institute of Immunology Co., Ltd., and supplied at 6 or 7 weeks of age. The rats were maintained in cages (≤ 3 animals per cage) and had free access to chlorinated water and food (FR-2, Funabashi Farm Co., Ltd.). The animal quarters were set at 23 ± 2 °C (allowable range: from 18 to 28 °C), humidity of 55 ± 10% (allowable range: from 30 to 70%), and a 12 h lighting cycle (lights on from 7:00 to 19:00). The acclimation period was for > 3 days.

### Tested compounds

rt-PA (ACTIVACIN for Injection) was purchased from Kyowa Hakko Kirin Co., Ltd. rt-PA was dissolved in adjunctive injection solvent and physiological saline (Otsuka Pharmaceutical Factory Inc.) for further dilution. The saline served as the control solution. The rt-PA was intravenously administered with a 1/10 vol. of bolus followed by 1 h of infusion using an infusion pump (TE-361, TERUMO Corporation) at doses of 1 or 10 mg/kg.

### Hematology

Hematological analysis was carried out to assess hematological abnormality. Blood was collected into a syringe containing 10 vol% of 3.13% sodium citrate via the abdominal aorta under sodium thiopental anesthesia. Hematological assessment (red blood cells, hemoglobin, hematocrit, platelets, white blood cells, neutrophils, lymphocytes, monocytes, eosinophils, and basophils) was performed using an automated hematology analyzer XT-2000iV (Sysmex Corporation).

### Basal tPA mRNA expression

To confirm whether tissue distribution of the tPA was conserved among strains, basal mRNA expression of tPA was evaluated. Five organs (liver, lung, brain, kidney, and heart) were excised from rats anesthetized using sodium thiopental after perfusion of 10 ml of physiological saline (Otsuka Pharmaceutical Factory Inc.) via the left ventricle. Total RNA extraction and reverse transcription was performed with an RNeasy Mini Kit (QIAGEN K.K.) and High Capacity cDNA Reverse Transcription Kit (Life Technologies, Inc.). Quantitative PCR was performed in absolute quantification using the 7900HT Fast Real Time PCR System (Applied Biosystems Inc.), Taqman Gene Expression Master Mix (Life Technologies, Inc.) and gene specific probes (Taqman Gene Expression Assays, Assay ID of rat tPA: Rn01482578_m1, β-actin [internal control]: Rn00667869_m1). All the procedures were conducted in accordance with the manufacturers’ instructions.

### Brain tPA expression after permanent focal cerebral ischemia

To compare tPA mRNA induction against pathophysiological stimuli between strains, a rat model of permanent focal cerebral ischemia was tested. Rats were treated with buprenorphine (0.04 mg/kg, subcutaneous, Otsuka Pharmaceutical Co., Ltd.) for analgesia and anesthetized with isoflurane. Focal cerebral blood flow (CBF) around the middle cerebral artery (MCA) was monitored by placing the probe of a laser Doppler flowmeter (ALF21D, ADVANCE Co., Ltd.) between the temporal muscle and the skull above the origin of the MCA. A nylon suture (4–0 fine MCAO suture L56PK10, Doccol Corporation) was inserted into the internal carotid artery (ICA) from the external carotid artery (ECA) to block blood flow into the MCA [25]. Focal cerebral ischemia was confirmed with the reduction of the laser Doppler flow signal. The nylon suture was placed permanently until sacrifice, and rats were housed in home cages after closure of incision and recovery from anesthesia. Brains were excised 24 h after the ischemia, and both ipsilateral and contralateral tissue was used for quantitative PCR in each animal.

### Coagulation and fibrinolytic parameters

To assess basal tPA concentration and other coagulation and fibrinolytic parameters, plasma or serum analyses were carried out. Citrated plasma was collected with centrifugation of the blood sample at 2200×*g* at 4 °C for 10 min. For serum collection, blood was collected via the jugular vein into a serum separation kit (FUCHIGAMI KIKAI, Y.K.), incubated at room temperature for 30 min, and centrifuged at 2200×*g* at 4 °C for 10 min. Prothrombin time (PT) and plasma fibrinogen concentration were measured using a HemosIL PT Fibrinogen HS PLUS and activated partial thromboplastin time (aPTT) was measured using a HemosIL SynthASil (LSI Medience Corporation) on a hemostasis analyser ACLTOP500 CTS (Instrumentation Laboratory), respectively. Plasma tPA concentration and α_2_-plasmin inhibitor (α_2_-PI) activity were determined with a Tissue Plasminogen Activator Rat ELISA Kit (Abcam plc.) and TESTTEAM S APL (SEKISUI MEDICAL CO., Ltd.), respectively. Free PAI-1 and plasminogen concentration were determined with a PAI1 (SERPINE1) Rat SimpleStep ELISA Kit (Abcam plc.) and a Plasminogen rat ELISA Kit (Abcam plc.), respectively.

### Tail bleeding model

To assess whether basal tPA increase affects normal hemostasis, bleeding time was assessed in a rat model of tail bleeding. Rats were anesthetized with thiopental sodium, and put on heating pads at approximately 37 °C to maintain their body temperature. An incision was made (depth 1 mm) with a blade (FAS-10, FEATHER Safety Razor Co., Ltd.) on the artery of the ventral part of the tail at 4 cm from the tip, and the blood was blotted every 30 s with filter papers (No. 2, Advantec Toyo Kaisha, Ltd.) for 30 min. The bleeding time was defined as the multiplication of the number of detectable blood stains on the opposite side of the filter paper that touched the blood by 30 s.

### Tissue factor-induced hypercoagulation model

To assess whether fibrinolytic activity was upregulated in another pathological model, plasma concentration of D-Dimer was determined in a TF-induced hypercoagulation model. Rats were anesthetized with sodium thiopental. Tissue factor (TF, Dade Innovin, Siemens AG, GTN-200A, 10 ml vial) solution was prepared by the addition of 5 ml of saline, and continuously administered to the rats via the jugular vein over a period of 20 min using the infusion pump (TE-361) set at 7.5 ml/kg/h. Blood samples (400 μl per time point) were collected from the jugular vein into a syringe containing 10 vol% of 3.13% sodium citrate before and 20, 45, 90, and 120 min after the TF administration. Citrated plasma was collected with centrifugation of the blood sample at 2200×*g* at 4 °C for 10 min. The plasma samples were diluted 20 times with factor diluent (LSI Medience Corporation) and then D-Dimer concentration, which is an indicator of fibrin (not fibrinogen) degradation, was measured using LPIA-ACE D-D dimer II (LSI Medience Corporation) on the ACL TOP 500 CTS.

### Dose-response of rt-PA in a thromboembolic stroke model

Blood was collected from the aorta of an anesthetized rat into a syringe containing 3.13% sodium citrate. The recombinant TF (10 ml vial) solution was prepared by adding 5 ml of saline. The whole blood (550 μl) was mixed with the TF (25 μl) in a 2-ml microcentrifuge tube and immediately drawn into a polyethylene tube (PE50, Becton, Dickinson and Company). The mixture was incubated at room temperature for 2 h to form whole blood clot and stored in a refrigerator set at 4 °C. The day after clot preparation, the clot was washed in saline to remove uncoagulated erythrocytes, cut into 2 cm pieces in length, and then transferred into another catheter (SP31, Natsume Seisakusho Co., Ltd.) connected with a Hamilton syringe filled with saline. Rats were treated with buprenorphine (0.04 mg/kg, s.c.) for analgesia and anesthetized with isoflurane. Focal CBF around the MCA was monitored by placing a probe of the laser Doppler flowmeter between temporal muscle and the skull above the origin of the MCA. The SP31 tube containing a piece of clot was inserted into the ICA from the ECA through the bifurcation of common carotid artery. The clot was injected with 40 μl of saline into the MCA territory of the anesthetized rat. If the CBF was not reached 40% or lower compared to that before the clot injection (100%), such animals were excluded and immediately euthanized with intravenous injection of thiopental at a dose of 100 mg/kg. Five minutes after the clot injection, rt-PA (1 or 10 mg/kg) or its vehicle (saline) was intravenously administered as a bolus (1/10 vol.) followed by infusion (9/10 vol.) via tail vein using the infusion pump for 1 h. Blood flow of the MCA was recorded for 110 min after the clot injection and expressed as percentage of mean CBF every 15 min against CBF before embolization. Area under the curve (AUC) of the CBF from 0 to 110 min after the clot injection (AUC_0−110 min_ [% * min]) was also calculated.

### Statistical analysis

Calculations were performed using Microsoft Excel 2010 (Microsoft Corporation). Data are expressed as the mean ± standard error of the mean (SEM). Statistical analyses were performed with a Student *t*-test or a Dunnett multiple comparison test using SAS System Release 9.2. (SAS Institute Inc.). Dose-dependency of the tested compounds was evaluated by Spearman’s rank correlation coefficient hypothesis testing using the SAS System Release 9.2. *P* values of < 0.05 were considered as statistically significant.

## Results

### Generation of tPA Tg rats

**Fig. 1:**
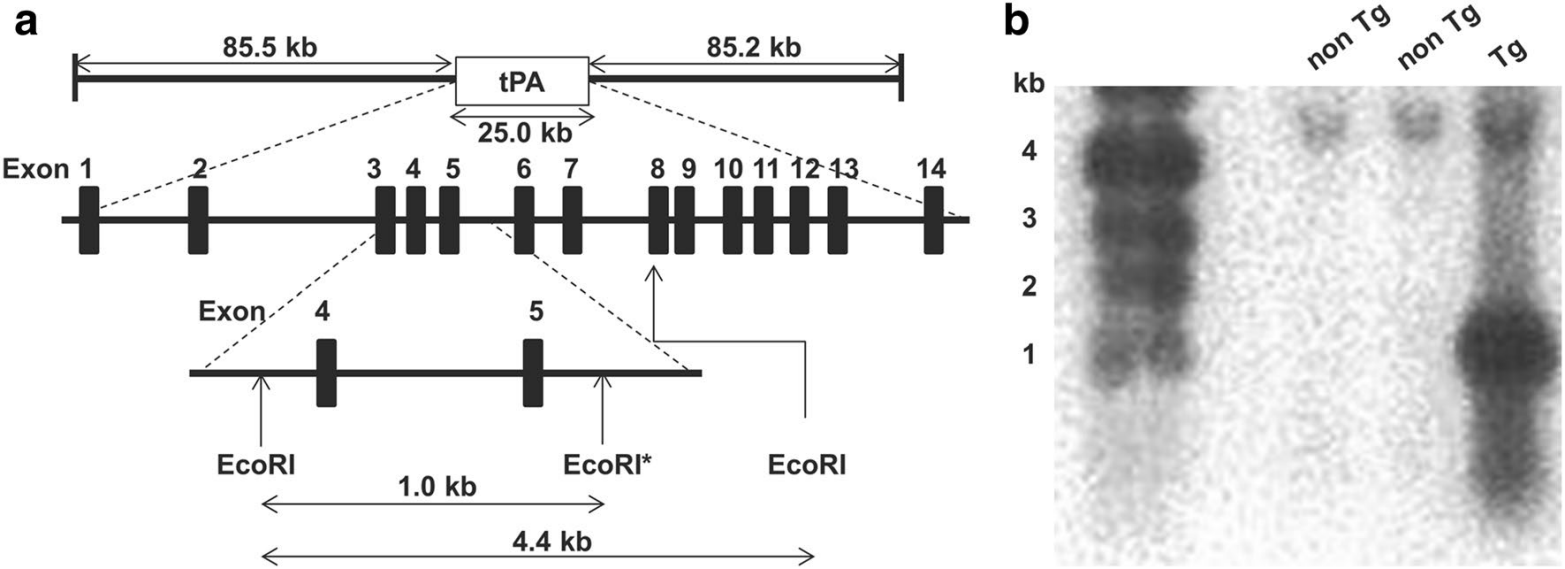
Analysis of the transgene structure in the genome of the tPA transgenic rats. **a** Schematic of transgene constructs and hybridization probe for Southern blot analysis. An EcoRI site (*) was created in the intron region downstream of Exon 5. **b** Southern blot analysis of tail DNA samples of the tPA Tg rats. Genomic DNA isolated from the tail was digested with EcoRI, electrophoresed through an agarose gel, and transferred to a nylon membrane. The nylon membrane was hybridized to the tPA probe to detect the 1.0 kb (transgene) and 4.4 kb (endogenous gene) restriction fragments

Three lines of tPA Tg rats were generated and analyzed by Southern blotting to confirm the presence of the transgene in genomic DNA. When rat DNA was digested with EcoRI and hybridized to the tPA probe described in “Materials and methods”, a 1.0 kb band was detected (Fig. 1a, b). One founder line was selected because of its high copy number (about ten copies) of tPA gene and its lack of detectable abnormal findings, including appearance, body weight, hematology, and systematization.

### Hematology

Hematological data are summarized in Table 1. There was no significant difference between the tPA Tg rats and the non Tg rats in hematological parameters, red blood cells, hemoglobin, hematocrit, platelets, white blood cells, neutrophils, lymphocytes, monocytes, eosinophils, and basophils, suggesting the tPA Tg rats have normal hematological feature.

**Table 1:** Hematological characterization in the tPA Tg and the non Tg rats

Parameter	non Tg	Tg	*P* value
	Mean ± SEM	Mean ± SEM	
RBC (× 10^4^/μL)	694.9 ± 6.3	693.6 ± 5.0	0.8790
HGB (g/dL)	13.1 ±0.1	12.8 ± 0.1	0.1311
Hematocrit (%)	36.9 ±0.4	36.5 ± 0.4	0.5192
Platelet (× 10^3^/μL)	846.3 ± 13.0	840.0 ±18.0	0.7759
WBC (× 10/μL)	410.2 ± 26.9	374.0 ± 25.5	0.3474
Neutrophil (× 10/μL)	59.7 ± 6.5	58.1 ± 3.4	0.8427
Lymphocyte (× 10/μL)	333.8 ± 23.6	300.5 ± 24.4	0.3437
Monocyte (× 10/μL)	11.4 ± 1.7	9.4 ± 1.1	0.3262
Eosinophil (× 10/μL)	5.3 ± 0.7	6.0 ± 0.4	0.4356
Basophil (× 10/μL)	0.0 ± 0.0	0.0 ± 0.0	N/A

Citrated whole blood was analyzed with an automated hematology analyzer. Data represent means ± SEM (n = 8 or 9). Statistical analyses were carried out by a Student *t*-test. A *P* < 0.05 was regarded as statistically significant

RBC: red blood cells, HGB: hemoglobin, WBC: white blood cells, N/A: not applicable

### Basal tPA mRNA expression

Basal mRNA abundance of tPA in five major organs (liver, lung, brain, kidney, and heart) is summarized in Table 2. In the five organs, tPA mRNA abundance was significantly upregulated in the tPA Tg rats, and its expression order among the organs was consistent (liver < lung < brain < kidney < heart) in the two strains.

**Table 2:** tPA mRNA abundance in the tPA Tg and the non Tg rats

Organ	non Tg	Tg	*P* value
	Mean ± SEM	Mean ± SEM	
Liver	1.00 ± 0.05	11.73 ± 0.71	< 0.0001
Lung	5.54 ± 0.28	28.76 ± 3.10	0.0003
Brain	6.02 ± 0.53	36.97 ± 3.08	< 0.0001
Kidney	10.79 ± 0.94	77.84 ± 7.53	< 0.0001
Heart	12.41 ± 1.68	89.62 ± 4.67	< 0.0001

tPA mRNA abundance of five organs (liver, lung, brain, kidney, and heart) was examined by quantitative PCR. The abundance was normalized with that of β-actin. The mRNA abundance was summarized as relative abundance of non Tg whose liver tPA mRNA expression was arbitrarily set to 1. Data represent means ± SEM (n = 4). Statistical analyses were carried out by a Student *t*-test. A *P* < 0.05 was regarded as statistically significant

### Brain tPA expression in permanent focal cerebral ischemia

**Fig. 2:**
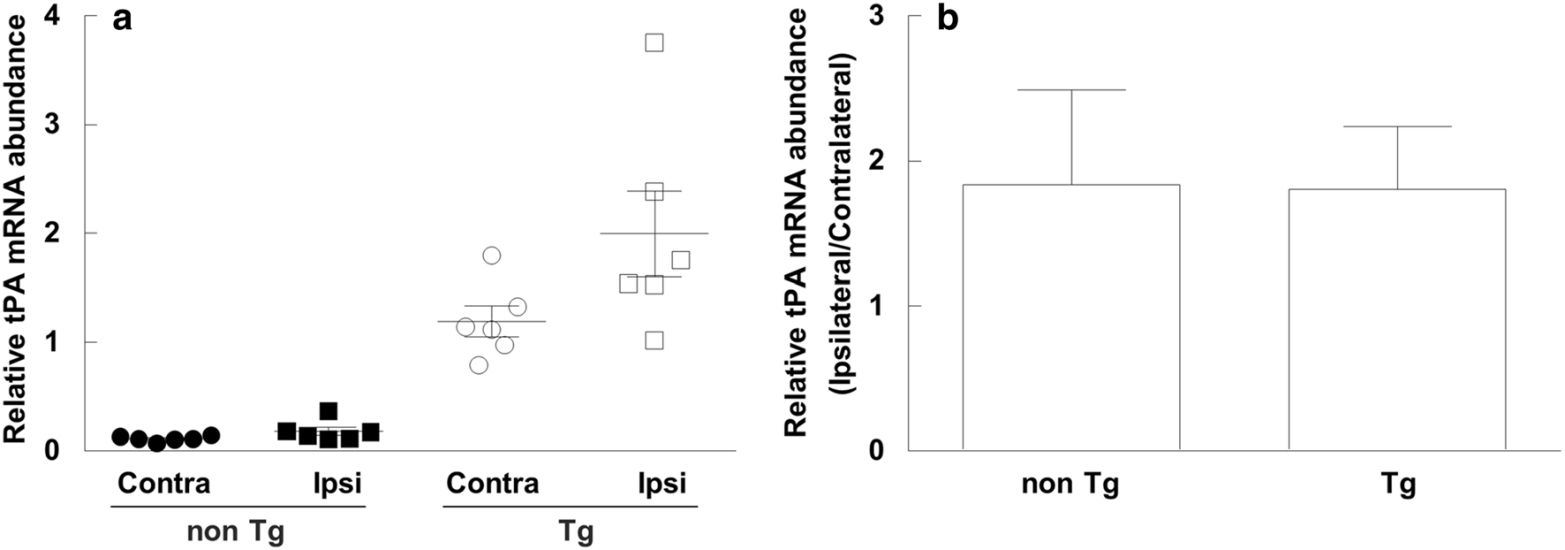
tPA mRNA induction stimulated with focal cerebral ischemia in the tPA Tg and the non Tg rats. A silicone-coated suture was permanently inserted into the internal cerebral artery to block the blood flow to the MCA of an anesthetized rat. Blood flow reduction of the MCA was monitored with a laser Doppler flowmeter. Rat brain was excised 24 h after the MCA occlusion and abundance of tPA mRNA in each brain hemisphere (ipsilateral and contralateral) was determined by quantitative PCR. **a** Individual values of contralateral or ipsilateral hemisphere in the two strains. **b** Post-ischemia tPA abundance calculated as ipsilateral/contralateral ratio. β-actin served as an internal control. Data represent means ± SEM (n = 6)

The CBF was decreased (12.5–30.7% compared with before embolization [100%]) in all animals subjected to focal cerebral ischemia. Brain tPA mRNA abundance after focal cerebral ischemia in the rats is shown in Fig. 2. The tPA mRNA abundance was increased in the ipsilateral side (2.00 ± 0.40 in the tPA Tg rats, 0.18 ± 0.04 in the non Tg rats) compared to the contralateral side (1.19 ± 0.14 in the tPA Tg rats, 0.11 ± 0.01 in the non Tg rats) (Fig. 2a), but the induction ratio (ipsilateral vs. contralateral) was comparable between the tPA Tg rats and the non Tg rats (1.81 ± 0.43 vs. 1.83 ± 0.66, Fig. 2b). No fatal cases were observed.

### Coagulation and fibrinolytic parameters

Coagulation and fibrinolytic parameters are summarized in Table 3. Plasma tPA concentration was significantly increased in the tPA Tg rats (0.229 ± 0.031 vs. 0.088 ± 0.009 ng/ml in the non Tg rats, *P* = 0.0004) and serum free PAI-1 was significantly decreased in the tPA Tg rats (625.9 ± 33.9 vs. 1395.8 ± 51.8 pg/ml in the non Tg rats, *P* < 0.0001). Slight shortening of aPTT was also observed in the tPA Tg rats (18.1 ± 0.4 vs. 19.5 ± 0.4 s in the non Tg rats, *P* = 0.0330). In contrast, other parameters, including PT, plasma α_2_-PI activity, serum plasminogen concentration, and plasma fibrinogen level, were not statistically significantly different between the two strains.

**Table 3:** Coagulation and fibrinolytic parameters of the tPA transgenic rats

Parameter	non Tg	Tg	*P* value
	Mean ± SEM	Mean ± SEM	
PT (s)	23.3 ± 0.3	23.8 ± 0.5	0.3892
aPTT (s)	19.5 ± 0.4	18.1 ± 0.4	0.0330
Plasma tPA (ng/mL)	0.088 ± 0.009	0.229 ± 0.031	0.0004
Serum free PAI-1 (pg/mL)	1395.8 ± 51.8	625.9 ± 33.9	< 0.0001
Plasma α_2_-PI activity (%)	183.7 ± 19.7	162.5 ± 6.2	0.3440
Serum plasminogen (μg/mL)	475.7 ± 22.6	464.9 ± 21.0	0.7327
Plasma fibrinogen (mg/dL)	283.2 ± 7.0	278.2 ± 3.5	0.5519

Serum plasminogen, serum free PAI-1, plasma tPA concentration, and α_2_-PI activity were determined with commercially available kits. PT, aPTT, and plasma fibrinogen were determined using an automated coagulometric analyzer. Data represent means ± SEM (n = 8 or 9). Statistical analyses were carried out by a Student *t*-test. A *P* < 0.05 was regarded as statistically significant

PT: prothrombin time, aPTT: activated partial thromboplastin time, tPA: tissue-type plasminogen activator, PAI-1: plasminogen activator inhibitor-1, α_2_-PI α_2_-plasmin inhibitor

### Tail bleeding model

**Fig. 3:**
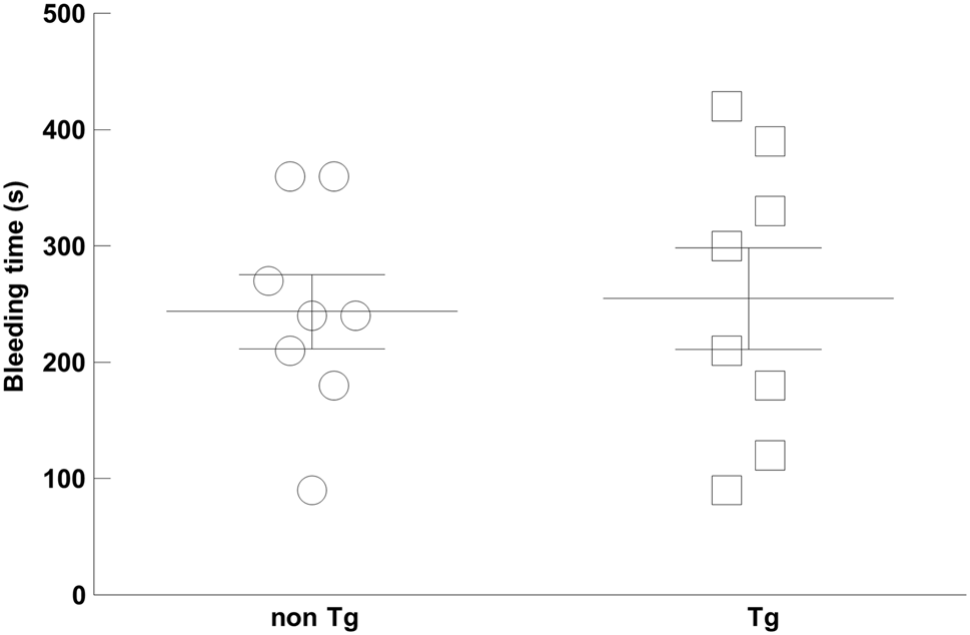
Bleeding profile of the tPA Tg rats in a tail bleeding model. Rats were anesthetized with thiopental sodium. An incision (1 mm in depth) was made on the artery of the ventral part of the tail at 4 cm from the tip, and the blood was blotted every 30 s with filter papers to measure bleeding time. Data represent means ± SEM (n = 8) with individual plots

Tail bleeding times in the bleeding model are shown in Fig. 3. There was no statistically significant prolongation of the bleeding time in the tPA Tg rats (255 ± 44 s in the tPA Tg rats vs. 244 ± 32 s in the non Tg rats).

### Tissue factor-induced hypercoagulation model

**Fig. 4:**
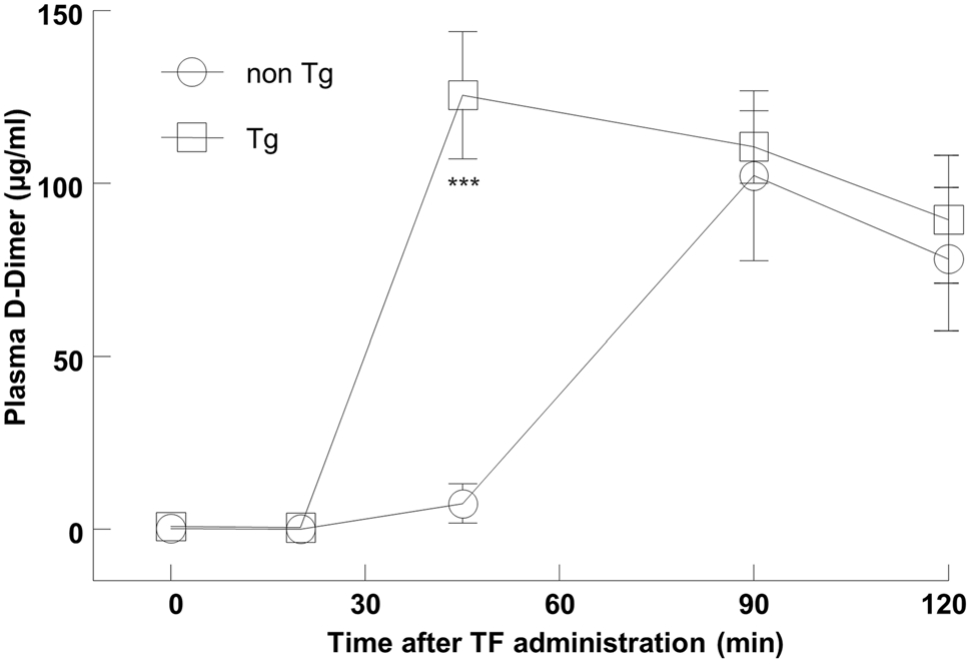
Profibrinolytic response of the tPA Tg rats in a tissue factor-induced thromboembolic model. Recombinant tissue factor (TF) was intravenously administered via the jugular vein using an infusion pump at a rate of 7.5 ml/kg/h for 20 min. Blood was collected from the jugular vein into a syringe containing 10 vol% of 3.13% sodium citrate at 0 (before) and 20, 45, 90, and 120 min after the TF administration. Plasma D-Dimer levels were determined as the biomarker of fibrinolysis. Data represent means ± SEM (n = 3 or 4). Statistical analyses were carried out by a Student *t*-test. A *P* < 0.05 was regarded as statistically significant. ***P = 0.0009 compared with the non Tg rats

Plasma D-Dimer concentration was measured as an indicator of fibrinolysis. Time-dependent change in D-Dimer is presented in Fig. 4. In the tPA Tg rats, statistically significant elevation of the plasma D-Dimer level was observed 45 min after the TF administration. There were not statistically significant changes in the plasma D-Dimer level at other time points (0, 20, 90, and 120 min after the TF administration). A non Tg rat was dead after 45 min blood sampling.

### Dose-response of rt-PA in thromboembolic stroke model

**Fig. 5:**
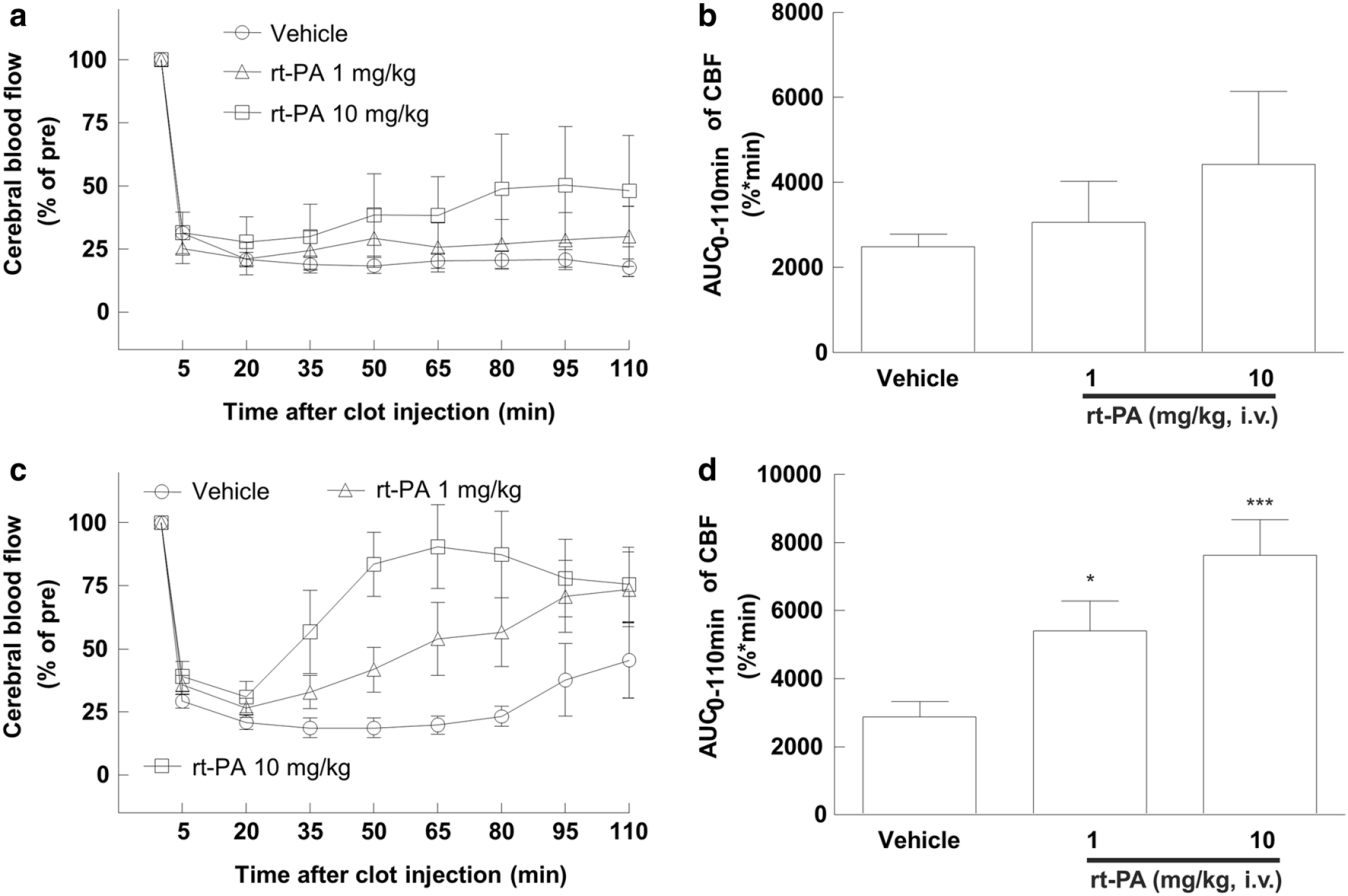
Comparison of dose response against rt-PA between the tPA Tg rats and the non Tg rats in a thromboembolic stroke model. Rat whole blood was coagulated in PE50 tubes using recombinant tissue factor to prepare whole blood clot. A piece of clot (2 cm in length) was injected into the MCA of an anesthetized rat. rt-PA (1 or 10 mg/kg) or its vehicle (saline) was intravenously administered as a bolus (1/10 vol.) followed by infusion (9/10 vol.) for an hour. Blood flow of the MCA was monitored with a laser Doppler flowmeter for 110 min after the clot injection and expressed as percentage of mean cerebral blood flow (CBF) against CBF before embolization (**a** for the non Tg rats and **c** for the tPA Tg rats). AUC_0−110 min_ of the CBF was also calculated (**b** for the non Tg rats and **d** for the tPA Tg rats). Data represents means ± SEM (n = 5–12). *P = 0.0402, ***P = 0.0005 compared with vehicle

Change in CBF and its area under the curve in the rat model of thromboembolic stroke is shown in Fig. 5. rt-PA at both doses did not increase the AUC of the CBF compared with the vehicle in the non Tg rats (2491.5 ± 291.3 in the vehicle, 3062.9 ± 957.9 in the rt-PA 1 mg/kg, and 4430.4 ± 1707.7 in the rt-PA 10 mg/kg, Fig. 5b). On the other hand, the AUC_0−110 min_ (%  * min) of the CBF was 2862.6 ± 461.5 in the vehicle group, 5397.8 ± 868.2 in the rt-PA 1 mg/kg group, and 7614.1 ± 1060.6 in the rt-PA 10 mg/kg group in the tPA Tg rats (Fig. 5d). Administration of rt-PA (1 and 10 mg/kg, i.v.) showed a dose-dependent (*P* < 0.0001) and statistically significant increase in the AUC_0−110 min_ compared with the vehicle (*P* = 0.0402 and *P* = 0.0005, respectively). One fatal case was observed just after the clot injection in the tPA Tg rats. Overall mortality rates were 3.22% and 0% in the tPA Tg rats and the non Tg rats, respectively.

## Discussion

To circumvent the species difference in the responsiveness to rt-PA between rats and humans, genetic manipulation of fibrinolytic factors in rats is one of the rational approaches. In the present study, we selected the rat tPA gene to generate transgenic rats to overcome the species difference since subtherapeutic supplementation of rt-PA has been carried out to evaluate TAFIa inhibitors, another class of fibrinolytic enhancers [8, 10, 11].

### Is the transgene expression under control of its endogenous promoter?

We generated for the first time tPA Tg rats that overexpressed tPA under the control of the endogenous transcriptional unit by using the BAC clone and the Red/ET system [24]. Southern blot analysis confirmed the presence of the transgene in genomic DNA. The levels of hematological parameters were comparable between the tPA Tg rats and the non Tg rats. In addition, tPA mRNA was significantly overexpressed in the tPA Tg rats and its expression order among five major organs was consistent (liver < lung < brain < kidney < heart) in the two strains. Since we introduced the endogenous transcriptional unit for tPA, induction against pathophysiological stimuli was expected to be conserved between the tPA Tg rats and the non Tg rats. To confirm this, we profiled the tPA mRNA induction by the permanent focal cerebral ischemia as a typical pathophysiological stimuli. Relative mRNA abundance (ipsilateral vs. contralateral) of tPA induced by the focal cerebral ischemia was comparable in the two strains, clearly indicating that the pathophysiological response of tPA expression is conserved in the tPA Tg rats.

It has been reported that the BAC transgenesis has several advantages over conventional transgenesis using short constructs with well-defined promoter regions in terms of reduced influence position effects, more highly conserved regulatory mechanisms of the transgene expression, and avoidance of mRNA surveilance mechanisms [26]. This approach seems to be highly reliable to control gene expression in vivo since BAC transgenesis have shown a success rate of 85% in reproducing an accurate and endogenous-like transgene expression pattern [27]. These previous findings are consistent with our observation, suggesting the BAC transgenesis is a powerful tool in rats as well as mice.

### Does the tPA Tg rat possess normal hemostasis?

The plasma tPA concentration was significantly increased and serum free PAI-1 was significantly decreased in the tPA Tg rats, demonstrating that basal production of tPA is also upregulated. Of importance, other parameters, including plasma α_2_-PI activity, serum plasminogen concentration, and plasma fibrinogen concentration, were equivalent between the two strains. These results suggest that increased tPA is neurtalized with abundant free PAI-1, and aberrant plasmin generation does not occur in the basal state. Furthermore, we confirmed that there was no statistically significant prolongation of bleeding time in the tPA Tg rats compared with the non Tg rats, indicating that hemostasis in the tPA Tg rats was normal. Taken together, the tPA Tg rats is not hyperfibrinolytic and possess normal hemostasis in spite of their basal tPA overexpression.

Previous reports have demonstrated that there are various biological effects of tPA beyond fibrinolysis such as LDL receptor related protein-dependent intracellular signaling that might lead to vascular leakage and hemorrhagic transformation [18–20]. In the present study, it seems that the extent of tPA overexpression in the tPA Tg rats do not affect vascular integrity since their bleeding time was comparable to that of the non Tg rats.

Hemostasis of the previous genetically modified mice of fibrinolytic systems such as PAI-1 knockout mice and α_2_-PI knockout mice was also reported to be equivalent to that of wild-type mice [21, 22]. So the tPA Tg rats seems to be similar to the two mice in terms of basal hemostatic profile.

### Does the tPA Tg rat show a mild fibrinolytic trait?

As mentioned above, both the PAI-1 knockout mice and α_2_-PI knockout mice do not present prolonged bleeding time. Despite their covert fibrinolytic activity in basal state, these mice show mild hyperfibrinolytic profiles in some pathological states such as lipoporysaccharide- or fibrin-injected models [21, 22]. In the present study, rats were subjected to the TF-induced thromboembolic model to assess fibrinolysis by measuring plasma D-Dimer, which is an indicator of fibrin (not fibrinogen) degradation [8, 10]. In the TF model, earlier increase in the plasma D-Dimer level was observed in the tPA Tg rats after the TF administration compared with the non Tg rats, demonstrating that the tPA produced in the tPA Tg rats was functional and showed the mild fibrinolytic trait under prothrombotic stimuli. Taking these baseline characteristics into account, we supposed that the utility of the tPA Tg rats is worth investigating for the pharmacological evaluation of rt-PA.

### Is the dosage of rt-PA brought closer to human stroke patients in the rat model of embolic stroke using the tPA Tg rats?

Lastly, we assessed whether the tPA Tg rats can minimize this gap in the effective dose of rt-PA in the thromboembolic stroke model. rt-PA (1 and 10 mg/kg, i.v.) showed a dose-dependent and statistically significant increase in the CBF compared with the vehicle in the tPA Tg rats, while it was not effective in the non Tg rats. These results suggest that the fibinolytic sensitivity of the tPA Tg rats to rt-PA might be closer to that of human stroke patients. This difference may be explained by the basal increase of the tPA concentration in the tPA Tg rats and in the whole blood clot employed to the rat stroke model, and/or ischemia-induced focal tPA induction. Other factors possibly influencing the dose-response of rt-PA are (i) physicochemical/histological clot compositions, and (ii) pharmacokinetics of intravenous rt-PA between the tPA Tg rats and the non Tg rats. The former factors had minimal impact on the dose-response since this was not changed even when blood clot injected was exchanged between strains (data not shown). Regarding pharmacokinetics, excretion pathway is important since rt-PA was administered intravenously. Previous studies have demonstrated that plasma half-life of rt-PA is very short (α phase: 1.18 min, β phase: 5.34 min) in rats [28], and mannose receptor and LDL receptor-related protein in liver was identified as responsible for its elimination. Taking plasma exposure of intravenously administered rt-PA (μg/mL order) [28] and endogenous tPA concentration (ng/mL order) into account, basal tPA concentration would be little impact on the excretion of rt-PA from circulation and plasma pharmacokinetic profile of rt-PA. Overall, The tPA Tg rats may therefore be of value to address the species difference in the responsiveness to tissue-type plasminogen activator (rt-PA) between wild-type rats and humans.

Several approaches can be considered to circumvent the species difference in the responsiveness to rt-PA between rats and humans. One of the possible approaches is creation or modification of novel or conventional rat stroke models. For example, Tomkins et al. reported that rt-PA showed statistically significant recanalization of blood flow in a rat model of carotid occlusion with doses of 4.5 mg/kg or more [16]. This is intriguing in that the disease model is characterized with naturally forming platelet-rich thrombi in the lumen of carotid artery [29]. However, there might be several consideration points on the plausibility of mimicking human stroke and responsiveness of rt-PA. In general, recanalization rates are higher in MCA occlusion than in carotid occlusion in human patients [30]. In addition, clot composition also affects fibrinolytic susceptibility: fibrin-rich thrombi is more easily dissolved than the platelet-rich thrombi [31]. Furthermore, the most popular stroke subtype indicated for rt-PA is cardioembolic stroke, characterized by MCA occlusion and fibrin-rich thrombi [32, 33], suggesting these factors should be taken into account to establish animal models of stroke. In these respects, we believe that our stroke model, MCA occlusion with a piece of fibrin-rich whole blood clot using the tPA Tg rats, is much closer to human stroke in terms of the responsiveness of rt-PA compared with the non Tg rats.

Regarding outcome measure, we selected CBF around the MCA. Direct and continuous measurement of the CBF enabled us to monitor precise pharmacodynamic effect of rt-PA. Early recanalization is the most important predictor of neurological outcome in rats and human stroke patients, and rt-PA and mechanical thrombectomies are indicated in patients with acute ischemic stroke with excellent outcomes [2, 34, 35]. Based on such a body of nonclinical and clinical evidences, we think that CBF measurement is enough for outcome measure in the rat stroke model since the purpose of the present study is to assess whether we can minimize the gap in the effective dose of rt-PA between rats and humans by using the tPA Tg rats.

### Experimental limitations

There are several experimental limitations in the present study. Firstly, we have not confirmed dose response relationship in the copy numbers of the transgene. Therefore, there would be more optimal copy numbers to circumvent the species difference. Secondly, timing of rt-PA administration (5 min after the clot injection) in the stroke experiment was earlier compared with therapeutic time-window in human stroke patients (< 4.5 h from onset of stroke). Since we measured the CBF before and after clot injection to confirm MCA occlusion, starting rt-PA administration 5 min after the clot injection would be allowable to be regarded as an example of therapeutic intervention. Therapeutic time-window analysis of rt-PA would be more informative. Thirdly, cut-off CBF value for the prediction of better neurological outcomes have not determined in our animal model since simultaneous evaluation of CBF and mortality/neurological outcome is difficult in our experimental design since continuous anesthesia (> 110 min) along with ischemia had affected morbidity within operative day. Determination of the cut-off CBF value would be of value to address the usefulness of the tPA Tg rats in preclinical stroke research.

In conclusion, we generated tPA Tg rats, which possessed the following targeted profile: (i) a conserved pathophysiological response of fibrinolysis, (ii) normal hemostasis, and (iii) effective doses of rt-PA in a thromboembolic stroke are closer to those of human patients. To our knowledge, this is the first report on generating tPA Tg rats and this might be beneficial for the pharmacological and toxicological evaluation of rt-PA. In addition, this animals are also applicable for the evaluation of other various fibrinolytic enhancers, such as tPA derivatives, plasmin derivatives, SMTP compounds, and TAFIa inhibitors.
