# Correction to: Generation and characterization of tissue-type plasminogen activator transgenic rats

**DOI:** 10.1007/s11239-017-1598-6

**Published:** 2017-12-16

**Authors:** Yusuke Ito, Kengo Noguchi, Yoshiyuki Morishima, Kyoji Yamaguchi

**Affiliations:** 1 Rare Disease & LCM Laboratories, Daiichi Sankyo Co., Ltd., 1-2-58 Hiromachi, Shinagawa-ku, Tokyo, 140-8710 Japan; 2 Pharmacovigilance Department, Daiichi Sankyo Co., Ltd., Tokyo, Japan; 3 Medical Science Department, Daiichi Sankyo Co., Ltd., Tokyo, Japan

## Correction to: Journal of Thrombosis and Thrombolysis 10.1007/s11239-017-1582-1

In the original publication of the article, the sentence in “Result” section have been incorrectly published as: “Three lines of tPA Tg rats were generated and analyzed by Southern blotting to confirm the presence of the transgene in genomic DNA. When rat DNA was digested with EcoRI and hybridized to the tPA probe described in “Materials and methods”, a 1.0 kb band was detected (Fig. 1a, b). One founder line was selected because of its high copy number (about ten copies) of tPA gene and itansgene) and 4.4 kb (endogenous gene) reding appearance, body weight, hematology, and systematization.”

The corrected sentence should read as: “Three lines of tPA Tg rats were generated and analyzed by Southern blotting to confirm the presence of the transgene in genomic DNA. When rat DNA was digested with EcoRI and hybridized to the tPA probe described in “Materials and methods”, a 1.0 kb band was detected (Fig. 1a, b). One founder line was selected because of its high copy number (about ten copies) of tPA gene and its lack of detectable abnormal findings, including appearance, body weight, hematology, and systematization.”

The original article has been corrected.

